# Absence of *BRAF* gene mutations in uveal melanomas in contrast to cutaneous melanomas

**DOI:** 10.1038/sj.bjc.6600919

**Published:** 2003-04-29

**Authors:** S C Edmunds, I A Cree, F Dí Nícolantonío, J L Hungerford, J S Hurren, D P Kelsell

**Affiliations:** 1 Centre for Cutaneous Research, Barts and the London School of Medicine and Dentistry, Queen Mary, University of London, 2 Newark Street, Whitechapel E1 2AT, UK; 2 Translational Oncology Research Centre, Queen Alexandra Hospital, Portsmouth PO6 3LY, UK; 3 Department of Pathology, Institute of Ophthalmology, Bath Street, London EC1V 9EL, UK; 4 Ocular Oncology Service, St Bartholomew's and Moorfields Eye Hospital, City Road, London EC1V9EL, UK; 5 Department of Surgery, Queen Alexandra Hospital, Portsmouth PO9 6AH, UK

**Keywords:** melanoma, BRAF, DNA sequence, mutation

## Abstract

The recent discovery of activating mutations in the *BRAF* gene in many cutaneous melanomas led us to screen the genomic sequence of *BRAF* exons 11 and 15 in a series of 48 intraocular (uveal) melanomas, together with control samples from three cutaneous melanomas and the SK-Mel-28 cell line, which has a *BRAF* mutation. The same mutation was detected in two-thirds of our cutaneous melanoma samples, but was not present in any uveal melanomas. This finding further underlines the distinction between uveal and cutaneous melanomas, and suggests that BRAF inhibitors are unlikely to benefit patients with uveal melanoma.

Uveal melanoma is the most frequent primary intraocular tumour in Caucasian adults, having an annual incidence rate of 0.7 per 100 000 people (Prescher *et al*, 1996). The eye is the most common site for noncutaneous melanomas, accounting for approximately 80% of such lesions (Sisley *et al*, 2000) and accounting for 13% of all deaths from melanoma because of its very high mortality rate (Albert *et al*, 1992). Both uveal and cutaneous melanomas originate from the melanocyte, but little is known about the underlying molecular pathogenesis of uveal melanoma. This is in contrast to cutaneous melanoma where there have been more substantial advances in detecting mutations (Chin *et al*, 1998). Both tumours differ significantly in their aetiology, with UV light appearing to play little or no part in the causation of uveal melanoma, unlike skin melanoma (Dolin *et al*, 1994). Uveal melanomas spread haematogenously leading to liver metastasis, whereas cutaneous melanoma spreads mainly via the lymphatics (Seftor *et al*, 1999) with skin metastases a more common problem. Unlike cutaneous melanoma, no genes or tumour-suppressor pathways have so far been convincingly linked to uveal melanoma (Edmunds *et al*, 2002).

It has recently been reported that a large proportion of cutaneous melanoma tumours contain activating oncogenic mutations in the *BRAF* gene (Davies *et al*, 2002). This is an oncogene in the RAS–RAF–MEK–ERK–MAP kinase pathway that mediates cellular response to growth signals. Genetic alterations to key components of this pathway are known to contribute to the development of many cancers (Pollock and Meltzer, 2002). Activating *RAS* point mutations are known to be found in more than 30% of human tumours, predominantly pancreatic, colonic, and in up to 36% of cutaneous melanomas (Demunter *et al*, 2001). *BRAF* is a gene that is regulated by RAS binding, and was shown to have missense mutations in 66% of primary melanoma tumours, 59% of melanoma cell lines, and 80% of melanoma short-term cultures (Brose *et al*, 2002; Davies *et al*, 2002). Mutations have also been detected in up to 82% of cutaneous melanocytic nevi (Pollock *et al*, 2003). Activation of this pathway has been noted in uveal melanoma tumours although mutations have not been detected in any of the *RAS* genes (*H*-, *K*, and *N*-*RAS*) (Mooy *et al*, 1991; Soparker *et al*, 1993). This makes *BRAF* an interesting candidate gene to screen in uveal melanoma tumours because of *BRAF* mutation being a potential mechanism for the activation of this pathway, and the fact that *BRAF* mutations are not thought to be related to the effects of UV light (Davies *et al*, 2002).

*BRAF* mutations were predominantly found in two small regions of the kinase domain of the BRAF molecule. The majority of the mutations were a single T→A base substitution at nucleotide 1796 in exon 15 of the *BRAF* gene, and in some of the adjacent codons. A smaller number of mutations were also found in a region of exon 11, and other lower levels of mutations have been reported in these codons in cancers including ovarian, sarcomas, lung (Brose *et al*, 2002), and colorectal tumours (Rajagopalan *et al*, 2002).

## MATERIALS AND METHODS

We screened the genomic sequence of *BRAF* exons 11 and 15 in a series of 48 uveal melanoma tumours, using primers taken from Davies *et al* (2002). DNA was extracted from tumours removed from enucleated eyes from Moorfields Eye Hospital as described previously and used in a previous study (Edmunds *et al*, 2002). All tumour samples were removed as part of patient treatment and with local ethical committee approval for use of the tissue in this study, and the study protocol adhered to the tenets of the Declaration of Helsinki. The median age of the patients was 61 years old, with a small bias towards male subjects (56%). These tumours were predominantly choroidal (74% of tumours), with smaller numbers of ciliary body (14%), and mixed choroidal–ciliary body (12%) type. We also tested DNA from three cutaneous melanoma metastases.

Polymerase chain reaction (PCR) products were amplified using Bioline Taq (Bioline, London, UK) in the following conditions: 5 min 96°C initial denaturation, 96°C 30 s, 55°C 1 min, 72°C 30 s for 30 cycles, followed by a final extension cycle for 5 min at 72°C (Hybaid, Ashford, UK). Polymerase chain reaction products were purified using a QIAquick PCR purification kit (QIAGEN, Crawley, UK). Purified PCR products are then directly sequenced using Big-Dye terminator chemistry and analysed on an AB Biosystems 377 automated sequencer (AB, Warrington, UK). As a positive control, the cutaneous melanoma cell-line SK-MEL-28 DNA was used, that was known to contain the exon 15 T1796A (V599E) mutation (Davies *et al*, 2002). As a negative control, blood DNA from several unaffected individuals was used.

Sequences were compared to the assumed wild-type sequence from the nontumour DNA, and to the human *BRAF* sequence (Genbank accession number: GI:179532). Sequences were aligned with wild-type sequence traces and compared by eye. Particular attention was given to the sequence around the two small regions of the kinase domain of the *BRAF* molecule located in exons 11 and 15 that contain all of the published mutations.

## RESULTS

**Figure 1:**
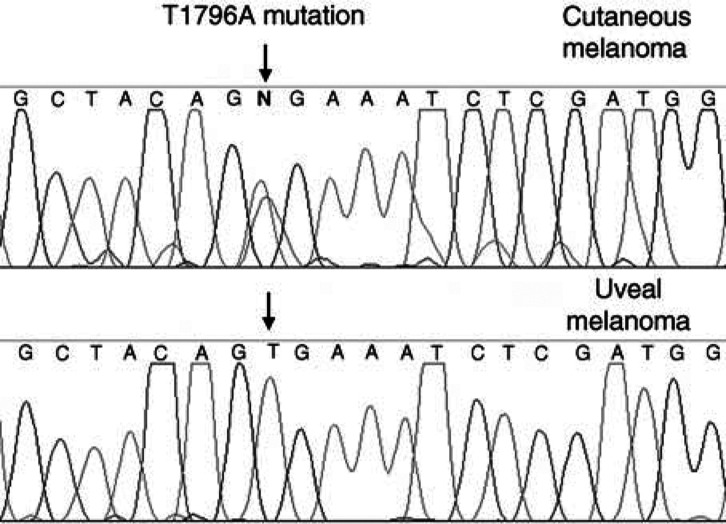
Sequencing trace showing the *BRAF* exon 15 T1796A (V599E) mutation in cutaneous melanoma DNA and wild-type sequence in a uveal melanoma tumour.

The SK-MEL-28 cell-line exon 15 T1796A (V599E) mutation was detected by sequencing, and the same mutation was also detected in two-thirds of the skin melanoma tumours studied. This result was expected as Davies *et al* (2002) had shown that 66% of the malignant melanoma tumours screened had *BRAF* mutations, and predominantly the T1796A mutation. In contrast to this finding, we could not detect exon 11 or 15 *BRAF* mutations in any of the uveal melanoma tumours screened. We were able to produce high-quality sequencing to screen for mutations in 35 samples for exon 15 and 23 samples for exon 11 (Figure 1

).

## DISCUSSION

It is possible that, with direct sequencing, low levels of mutation could have been missed, as it is not as sensitive a technique as SSCP or DHPLC. Our tumour DNA samples were very pure though, and there was unlikely to be any contamination from normal tissues. Sequencing would not be able to detect mutant alleles present at low frequency because of somatic mosaicism, but the significance of very low levels of mutant tumour cells would be questionable. It is also possible that there were mutations in other areas of the *BRAF* gene, as we only screened exons 11 and 15, and predominantly concentrated on the mutated hotspots. All previously reported mutations have been concentrated to these two hotspot regions in the *BRAF* kinase domain, hence, mutations in different regions of the molecule are unlikely to be able to activate the oncogene in such a strong manner.

Here, we show another potentially important cancer-associated gene that is not mutated in sporadic uveal melanoma. Many other studies have found significant genetic (Naus *et al*, 2000; Soufir *et al*, 2000; Edmunds *et al*, 2002) and cytogenetic differences (Sisley *et al*, 2000) between the tumour types, despite both cells originating from the same cell type. Uveal melanoma is less studied in comparison to cutaneous melanoma, but to date no significant levels of mutated tumour-suppressor genes have so far been convincingly linked to it (Edmunds *et al*, 2002). Epigenetic mechanisms of gene inactivation may play a more important role in this tumour. If the RAS/RAF pathway is activated in uveal melanoma, then it is unlikely to be because of activating mutations in *RAS* or *B-RAF*, but other members of this pathway have yet to be studied, including *A-RAF*, *C-RAF* (*RAF1*), and *GAP1*.

Our findings further highlight the fact that cutaneous and uveal melanomas are very different tumours, and that the oncogenesis of uveal melanoma uses very different mechanisms and genes to cutaneous melanoma. This has implications for treatment, as BRAF inhibitors are now undergoing clinical trials from which uveal melanoma patients are unlikely to benefit. (Chow *et al*, 2001; Coudert *et al*, 2001; Rudin *et al*, 2001; Cripps *et al*, 2002).
